# The Cost and Impact of the Interim Federal Health Program Cuts on Child Refugees in Canada

**DOI:** 10.1371/journal.pone.0096902

**Published:** 2014-05-08

**Authors:** Andrea Evans, Alexander Caudarella, Savithiri Ratnapalan, Kevin Chan

**Affiliations:** 1 Department of Paediatrics, The Hospital for Sick Children, University of Toronto, Toronto, Ontario, Canada; 2 Department of Family Medicine, St. Michael's Hospital, University of Toronto, Toronto, Ontario, Canada; 3 Dalla Lana School of Public Health, University of Toronto, Toronto, Ontario, Canada; 4 Department of Paediatrics, Eastern Health, Memorial University, St. John's, Newfoundland, Canada; University of Louisville, United States of America

## Abstract

**Introduction:**

On June 30, 2012, Interim Federal Health Program (IFHP) funding was cut for refugee claimant healthcare. The potential financial and healthcare impacts of these cuts on refugee claimants are unknown.

**Methods:**

We conducted a one-year retrospective chart review spanning 6 months before and after IFHP funding cuts at The Hospital for Sick Children, a tertiary care children's hospital in Toronto. We analyzed emergency room visits characteristics, admission rates, reasons for admission, and financial records including billing from Medavie Blue Cross.

**Results:**

There were 173 refugee children visits to the emergency room in the six months before and 142 visits in the six months after funding cuts. The total amount billed to the IFHP program during the one-year of this study was $131,615. Prior to the IFHP cuts, 46% of the total emergency room bills were paid by IFHP compared to 7% after the cuts (p<0.001).

**Interpretation:**

After the cuts to the IFHP, The Hospital for Sick Children was unable to obtain federal health coverage for the vast majority of refugee claimant children registered under the IFHP. This preliminary analysis showed that post-IFHP cuts healthcare costs at the largest tertiary pediatric institution in the country increased.

## Introduction

Canada signed the 1951 Convention Relating to the Status of Refugees, which defines the rights of refugees, refugee claimants and the obligations of states [1]. In 1957, Canada created the Interim Federal Health Program (IFHP) (Order-in-Council PC 157-11/848), which provided funding for refugees and refugee claimants for medications, vaccines, periodic health assessments, psychological services and dental care. On June 30^th^, 2012, the federal government repealed the IFHP, claiming that IFHP cuts would reduce health care costs [2], [3].

In the post-IFHP cuts system, a portion of refugee applicants, including those who are from a Designated Country of Origin (DCO), are no longer entitled to any coverage or primary care services [4], [5]. Refugee claimants can either continue to receive the same funding for services previously offered; receive funding for services deemed “urgent” or “essential”; or some groups, for example those from a DCO, receive no government funding for health care [6]–[7]. Thus, certain refugee populations with significant morbid conditions, such as diabetes, pregnancy and birth, mental health, and sexual abuse, may have little funding for healthcare [7]. To protect the Canadian public, the federal government stated that all refugee claimants would be covered for the prevention or treatment of “a disease that is a risk to public health or to treat a condition of public safety concern” [7]. Unfortunately, the funding cuts on June 30^th^, 2012 increased the “confusion and administrative complexity” preventing refugee claimants from accessing health care, even if they have a disease of public health concern [6].

Immigrants and refugees face barriers to accessing health care. [8]–[13]. There is concern that the changes in healthcare coverage will lead to a worsening of the health disparities in already vulnerable populations, worsen health outcomes and increase health care costs [14]. The Canadian Medical Association, many national medical organizations, and a number of provincial governments have publicly asked the federal government to repeal these policy changes [15]–[16].

There have been no systematic studies evaluating the impact of these policy changes since these cuts have been implemented. The objective of this study is to examine the impact of the funding changes to the IFHP at the Hospital for Sick Children (SickKids), specifically looking at the impacts on health care payments to the hospital, health care costs and changes in Emergency Room (ER) visits and hospitalization rates.

## Methods

### Study population

Our study included all emergency room (ER) visits at SickKids of children < 18 years, with payment status classified as IFHP from January 1, 2012 to December 31, 2012. The time period was chosen to capture the ER use 6 months before and after the funding cuts to the IFHP. Patients were classified as registered under the IFHP if they presented with IFHP papers, were refused claimants, refugee claimants ‘in process’ or pre-hearing. Patients were excluded from the database by SickKids if they were uninsured, undocumented, arriving without IFHP papers, permanent immigrant, temporary worker or otherwise not identified as IFHP refugee claimants in the emergency room database.

The child refugee claimant populations measured six months before and six months after the IFHP cuts were similar in terms of their migratory and legal status, with the exception of the institution of the Designated Country of Origin list that affected patients arriving to Canada after December 15, 2012. Only 5% of the population in our study arrived after this date, and none of these were on the DCO list. Furthermore, our population before and after the cuts were similar in terms of the number of refugees in Ontario with access to our hospital. The documented numbers of refugees settled in Canada was 24,981 in 2011 and 23,056 in 2012 [17].

All research in this article was approved by the institutional review board at The Hospital for Sick Children, in Toronto, Canada.

### Data Collection

The following information was extracted from the medical records: ER and admission data (including demographic data, migratory status categories, and severity of illness as measured by the Canadian Triage Acuity Score (CTAS)) was taken from the Wellsoft Emergency Department Information System. International Classification of Disease, 10^th^ Edition codes for most responsible diagnosis at admission and country of origin data were abstracted from the charts of patients who were admitted to hospital by two principal authors (AE and AC).

During the period of the study, SickKids did not significantly change its processes in response to the IFHP coverage changes. Billing information for ER visitors was taken from the Accounts Receivables database of SickKids, as of August 20, 2013.

Billing information included the amount billed to the non-profit insurance company Medavie Blue Cross, who provides health insurance coverage for the IFHP, and the amount paid from Medavie Blue Cross to SickKids.

### Data Analysis

Data was analyzed using SPSS (2012 v.21, Armonk, NY: IBM Corp). Statistical analysis used the Kolmogorov-Smirnov Test for normality of distribution. For variables with normal distribution, a student's t-test compared differences between two independent groups. Chi-squared test or Mann-Whitney U compared differences between two independent groups with non-normal distributions. Results were considered significant with a p-value <0.05, and a power of 0.80.

## Results

There were 173 documented visits by child refugee claimants under the IFHP program to the emergency room in the six months pre-IFHP funding changes and 142 visits in the 6 months post-IFHP changes. The number of children presenting to the ER in the same time period was 25,755 prior to the cuts and 31,189 after the cuts, where the proportion of refugees presenting to the ER after the cuts significantly decreased (p<0.01). Visit characteristics for refugee children pre- and post-IFHP cuts are similar (see Table 1). The high acuity visits (CTAS 1 or 2) represented 20% and 19% of visits pre- and post-IFHP changes, respectively. There was no significant difference between age, gender, length of stay, or CTAS score pre- and post-IFHP funding cuts or between the refugee children and the general population seen at SickKids.

**Table 1:** Emergency Room Visit Characteristics of Refugee Claimants Before and After IFHP Funding Cuts.

	Before the IFHP Cuts	After the IFHP Cuts	p-value
	n = 173	n = 142	
Mean Age (years)	6.8	6.8	0.87
Male (%)	47	58	0.07
CTAS 1 (%)	1.2	0.7	0.96
CTAS 2 (%)	19.0	18.3	
CTAS 3 (%)	41.0	40.1	
CTAS 4 (%)	36.0	37.3	
CTAS 5 (%)	2.3	3.5	
Admitted (%)	6.4	12.0	0.08
Admission Length of Stay (days)	2.8 [1.6–3.0]	2.2 [0.8–2.8]	0.32

Square brackets represent interquartile range.

After the implementation of funding cuts, the admission rate of refugee children increased from 6.4% to 12.0% (p = 0.08). The admission rate at SickKids for all patients pre-IFHP cuts was 11.1% and post-IFHP cuts was 10.0% (p = 0.90). The top three most responsible diagnoses by ICD-10 code for admission by refugee children during the study time period was sickle cell anemia with crisis (4), epilepsy (not intractable) (3), and appendicitis (2). There were no respiratory or viral illnesses in the child refugee population admitted. The most common reason for admission for the general population in the same time period was pneumonia, supracondylar fracture of the humerus, and sickle cell anemia with crisis.

The country of origin was known for 21 out of the 28 admissions. Six (29%) of these admissions were from patients of a country currently on the Designated Country of Origin list.

The billing data does not form a normal distribution (z  =  1.8 to 6.9, p <0.01). Bills from Medavie Blue Cross insurance were either not paid or fully paid (only 4% of paid bills were partially paid) (see Table 2). However, the total number of bills paid, and the total amount of money unpaid by Medavie Blue Cross insurance was significantly lower after the IFHP policy changes (p<0.01). Overall, 93% of the ER bills submitted by SickKids to Medavie Blue Cross insurance post-IFHP changes were unpaid, whereas 54% of the bills pre-IFHP changes were unpaid. The total admission bill in the six months post the IFHP cuts is higher and is influenced by one outlier admission bill.

**Table 2:** Emergency room and admission bills for refugee children to the Hospital for Sick Children 6 months before and 6 months after IFHP changes.

		Before IFHP Cuts (Jan 1 to June 30 2012)	After IFHP Cuts (July 1 to Dec 31 2012)	Total	p-value
		n = 173	n = 142	n = 315	
Emergency Room					
	Median Bill ($) *	93.70 [93.70–93.70]	93.70 [93.70–93.70]	n/a	
	Total Billed ($)	20,010	13,549	33,559	0.74
	Number of Bills Unpaid **	98 (57%)	131 (91%)	229 (73%)	<0.001
	Total Unpaid ($)	10,706 (54%)	12,601 (93%)	23,307	<0.001
Admissions					
	Median Bill ($)	1337,40 [668.60–2006.10]	1671.75 [668.70–4666.68]	n/a	
	Total Billed ($)	14,912	73,144	88,056	0.28
	Number of Bills Unpaid	7 (64%)	14 (82%)	21 (75%)	0.26
	Total Unpaid ($)	8,894 (60%)	31,483 (57%)	40,377	0.09
Total Billed ($)		34,922	86,693	131,615	0.33
					
Total Owing		19,600	44,084	63,684	<0.001

Square brackets represent interquartile range.

*Bills reported represent billing data for refugee children registered under the Interim Federal Health Program

**Partially paid bills are not included in this category.

There were 12 patients who changed billing categories between the March 28, 2013 and August 20^th^ 2013, when the Accounts Receivables database was audited. Of these patients, 6 changed from IFHP coverage to OHIP (provincial) coverage, while 6 changed from IFHP to uninsured.

## Interpretation

In 2012, 57% percent of Canadian refugee claimants entered in the province of Ontario, and Toronto was the city receiving the most refugee applicants in the country [17]. The data we present from Toronto's largest pediatric hospital is therefore of considerable value in assessing the impact of refugee health policy changes on child refugee claimants. Eight months after the last bill of this study, only 7% of ER bills were refunded by the IFHP. There is an increase in overall health care costs paid by SickKids (not reimbursed by Medavie Blue Cross), which suggests that the IFHP changes are leading to a downloading of the costs for refugee care to hospitals and therefore, provinces. Despite these increased bills, the total annual cost of caring for refugee children's emergency room and admitted stays at Canada's largest pediatric institution was only $131,615.

The IFHP system costs the Canadian people $50 million per year. These policy changes were projected to save taxpayers $20 million per year [4]–[5]. Cuts to the IFHP would result in cost savings if refugee children registered less often, and unregistered refugee children were to have their healthcare covered by an outside program (IFHP) or institution (hospital). However, the reality is that institutions like SickKids have put in place a policy to give health care to those who present to the emergency room. Thus, if a refugee child is no longer covered by the IFHP, and the refugee child's family cannot pay for the cost of the healthcare, the deficit is paid by the institution treating the patient. This provides healthcare savings at the federal level, but ultimately the cost is transferred to the institutions seeing the patient.

Furthermore, shifts in the levels of health care access (hospital to primary-based care or vice-versa) due to affordability and administrative hurdles may make the vulnerable refugee population sicker, eventually leading to overall increase in healthcare costs. Further studies are required to study shifts in healthcare access, and changes in accessibility of health care due to the change in IFHP coverage policies.

Prior Canadian evidence reveals that refugees are twice as likely to encounter difficulty in accessing care and had disproportionately lower self-reported health status compared to other immigrants [17]–[21]. The significant decrease in the amount of refugees presenting to the ER after the cuts were imposed may indicate that the cuts are adding to this difficulty in accessing care. Out of the admitted children where country of origin was known, only 29% of patients were from countries on the DCO list. This means that these patients would not receive covered health care even for emergent presentations following the IFHP changes.

One of the consequences accompanying the IFHP changes has been the confusion surrounding funding rules. The new multi-tiered program, and unspecified definitions of “essential” and “urgent” leaves many clinicians, administrators and patients confused as to who would be covered for what health care. Families with low socioeconomic status and limited health coverage often fear the impact of medical bills and delay seeking health care [6]. Institutions such as SickKids do not have the logistical capacity during emergencies to call Medavie Blue Cross prior to the assessment and treatment of a refugee child. Even if the procedure recommended by the government was feasible, there is evidence that approximately 90% of patients would be required to pay for their ER visits (the rate of unpaid bills currently). Certain institutions, and particularly smaller access points such as walk-in-clinics may not have the funding to cover costs, leaving it unknown how many would receive the care they needed.

This study is limited by the narrow range of data, relatively small number of patients, and experience of a single-institution. A sixth-month study period after the IFHP cuts was selected because this time period was prior to the institution of selection of refugees covered by the IFHP based upon country of origin (DCO list) and prior to there being a demonstrated shift in the population of in-country landed refugees. The documented numbers of refugees settled in Canada decreased from only 7% in 2011 to 2012 (24, 981 vs 23, 056) [17]. Since January 2013 (and the end of our study period), there has been a documented decrease by 70% of refugee applicants [17]. We recognize that the duration could have contributed to seasonality effects on admission and billing rates. However, none of the admissions by refugee claimants following the policy changes were linked to respiratory or viral illness, and the admission rate of the general population did not change for the same study period.

We acknowledge that confusion around the changes may have decreased the ability of patients to access health care. Certain refugees may have presented without valid IFHP papers, but this would probably lead to an underestimation of our results. We recognize that the number of children that were admitted is small and as such the increases in admission rate and admission costs cannot be interpreted without further studies. As such, future studies including multi-site studies may provide better information on the impact of these changes to refugee access to health care and characterize a shift of healthcare utilization (primary health care to hospital care).

After the cuts, over 90% of the bills were not reimbursed by the IFHP. Thus, the majority of refugee claimant children who were registered under the IFHP and treated at Sick Kids did not have their healthcare costs covered by the program. Contrary to the Canadian federal government statement that the IFHP program cuts would lead to overall cost savings, our study shows evidence of downloading of costs to the hospital level. Further population level studies would help elucidate if healthcare access is changing and if the illness severity of children presenting to hospitals have worsened following the IFHP program cuts.
