# Correction: The Cost and Impact of the Interim Federal Health Program Cuts on Child Refugees in Canada

**DOI:** 10.1371/journal.pone.0106198

**Published:** 2014-08-19

**Authors:** The PLOS ONE Staff

There is an error in Table 2. Under admissions, before IFHP CUTS, the Median Bill dollar amount should read 1337.40 not 1337,40. Please see the corrected Table 2 here.

**Table 2:** Emergency room and admission bills for refugee children to the Hospital for Sick Children 6 months before and 6 months after IFHP changes.

	Before IFHP Cuts (Jan 1 to June 30 2012)	After IFHP Cuts (July 1 to Dec 31 2012)	Total	p value
	n = 173	n = 142	n = 315	
*Emergency Room*				
Median Bill ($)*	93.70 [93.70-93.70]	93.70 [93.70-93.70]	n/a	
Total Billed ($)	20,010	13,549	33,559	0.74
Number of Bills Unpaid **	98 (57%)	131 (91%)	229 (73%)	<0.001
Total unpaid ($)	10,706 (54%)	12,601 (93%)	23,307	<0.001
*Admissions*				
Median Bill	1,337.40 [668.80-2006.10]	1,671.75 [668.70-4666.68]	n/a	
Total Billed ($)	14,912	73,144	88,056	0.28
Number of Bills Unpaid	7 (64%)	14 (82%)	21 (75%)	0.26
Total unpaid ($)	8,894 (60%)	31,483 (57%)	40,377	0.09
Total Billed ($)	34,922	86,693	**131,615**	0.33
Total Owing	19,600	44,084	**63,684**	<0.001

Square brackets represent interquartile range.

*Bills reported represent billing data for refugee children registered under the Interim Federal Health Program

**Partially paid bills are not included in this category.
